# Unusual Esophageal Foreign Body: A Table Fork

**DOI:** 10.1155/2013/987504

**Published:** 2013-03-24

**Authors:** Emilio Mevio, Niccolò Mevio

**Affiliations:** Department of Otorhinolaryngology, Ospedale Fornaroli via Donatore del Sangue, 20013 Magenta, Italy

## Abstract

The presence of an esophageal foreign body (EFB) is a medical emergency requiring urgent evaluation and treatment. Swallowing of foreign bodies is most common in children aged between 6 months and 6 years, in whom it usually occurs during games. In adults, foreign bodies tend to be ingested accidentally together with food. The authors report an unusual case of EFB (a table fork) in an adult and briefly report the clinical presentation and the therapeutic procedures adopted in this case and similar cases.

## 1. Introduction

The presence of an esophageal foreign body (EFB) is a frequent reason for emergency room visits. Patients complain of dysphagia, hypersalivation, regurgitation, odynophagia, neck pain, and retrosternal discomfort. A detailed anamnesis together with anteroposterior and lateral chest X-rays normally allows a rapid diagnosis.

In most cases, EFBs are found to be impacted in the upper part of the esophagus at the level of the first anatomical constriction.

The type of EFB differs depending on the age and eating habits of the subject. Thus, EFBs found in children are often coins, safety pins, toy parts, and small batteries [1], whereas in adults they are more likely to be boluses of food, meat bones or meat bone fragments, fish bones, parts of dentures, toothpicks, and nails or screws [2, 3].

In childhood, foreign bodies are usually ingested accidentally during games. In adulthood, the involuntary ingestion of foreign bodies is almost always correlated with the presence of predisposing factors, such as dentures, decreased sensitivity of the oropharyngeal mucosa, and neurological diseases. The presence of certain preexisting pathological conditions (e.g., strictures, diverticula, malignancy, and achalasia) can facilitate EFB impaction. 

## 2. Clinical Case

A 62-year-old Caucasian male entered the emergency room complaining of dysphagia, hypersalivation, and dyspnea. He was evidently drunk. He reported that he had been touching the roof of his mouth with the handle of a table fork in an attempt to stop persistent hiccups and in so doing had accidentally swallowed the fork.

**Figure 1:**
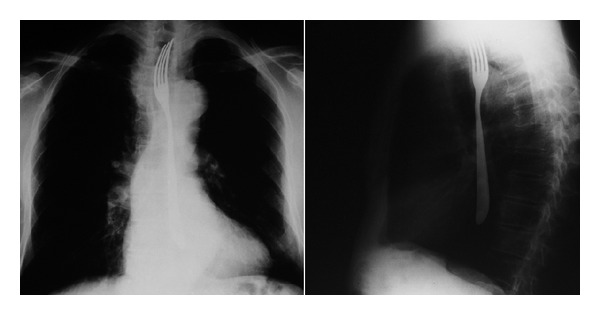
Antero-posterior and lateral X-rays of the thorax showing the foreign body (a table fork) occupying the entire thoracic esophagus.

Antero-posterior and lateral chest X-rays showed the fork in the esophagus (Figure 1). A fiber-optic endoscopic examination was performed and showed the prongs of the fork projecting from the upper esophageal sphincter in the retroarytenoid area.

The patient underwent general anesthesia during which the 22 cm long fork was removed under direct vision. The patient was discharged on the second day after surgery, after a swallow contrast study had shown a normal esophagus.

## 3. Discussion

The clinical approach to EFB depends on the type of material ingested and on the patient's symptoms and physical conditions. In about 80% of cases, an EFB will pass uneventfully through the gastrointestinal tract; endoscopy is performed in about 20% of cases and surgery in less than 1%. The only absolute indication for surgery is the presence of perforation [2].

An EFB can be removed by endoscopic approach, using either a rigid esophagoscope or a fiber-optic endoscopic instrument. Both methods are highly effective, and each has its advantages in certain situations. 

The first technique requires general anesthesia and has the main advantages of continuous visual control during the maneuvers and the possibility of extracting sharp foreign bodies safely, without damage to the esophageal mucosa. 

Flexible endoscopy has recently become the method of choice for the extraction of EFBs from the upper part of the digestive tract. In European countries, flexible endoscopy is six times more common than rigid endoscopy [4].

Only in cases presenting major complications, such as esophageal perforation or lesions of adjacent vessels, might a mediastinal surgical approach be necessary [5, 6]. 

The case reported is extremely rare. Only three cases of a fork in the esophagus were found in the literature [7–9]. Two of these patients, suffering from eating disorders, were trying to trigger vomiting using the handle of the fork to irritate their pharynx when the fork accidentally slipped into the esophagus [7, 8]. The circumstances of the most recent case are the same as those of the patient observed by us: an attempt to stop hiccups [9]. In all cases, the fork presented with the prongs pointing up and was removed under direct vision. This made the extraction maneuvers easier and reduced the risk of injury to the esophageal mucosa.

## References

[1] Rybojad B, Niedzielska G, Niedzielski A, Rudnicka-Drozak E, Rybojad P (2012). Esophageal foreign bodies in pediatric patients: a thirteen-year retrospective study. *ScientificWorldJournal*.

[2] Ambe P, Weber SA, Schauer M, Knoefel WT (2012). Swallowed foreign bodies in adults. *Deutsches Ärzteblatt International*.

[3] Soccorso G, Grossman O, Martinelli M (2012). 20 mm lithium button battery causing an oesophageal perforation in a toddler: lessons in diagnosis and treatment. *Archives of Disease in Childhood*.

[4] Schmidt H (2012). Foreign body in ENT medicine. *HNO*.

[5] Tersíp T, Simonek J, Pafko P (2002). Complications of endoscopic extraction of foreign bodies and their treatment. *Rozhledy v Chirurgii*.

[6] Wang S, Liu J, Chen Y, Yang X, Xie D, Li S (2013). Diagnosis and treatment of nine cases with carotid artery rupture due to hypopharyngeal and cervical esophageal foreign body ingestion. *European Archives of Oto-Rhino-Laryngology*.

[7] Katsas AG (1968). Ingested table fork. *Archives of Surgery*.

[8] Jones TM, Luke LC (1998). Life threatening airway obstruction: a hazard of concealed eating disorders. *Emergency Medicine Journal*.

[9] Abrão J, Khabbaz KM, Abrão JM, Coutinho DJ, Juliano EAC (2003). Unusual foreign body in the esophagus: a challenge for the anesthesiologist. *Acta Anaesthesiologica Scandinavica*.

